# YKT6 promotes breast cancer progression and is associated with poor prognosis and immune infiltration

**DOI:** 10.3389/fimmu.2026.1742661

**Published:** 2026-04-22

**Authors:** Meilin Zhang, Jingjing Yuan, Yaxuan Liu, Yiran Qiu, Mingdi Zhang, Hongliang Chen

**Affiliations:** Obstetrics & Gynecology Hospital of Fudan University, Shanghai Key Lab of Reproduction and Development, Shanghai Key Lab of Female Reproductive Endocrine Related Diseases, Shanghai, China

**Keywords:** bioinformatics, biomarker, breast cancer, immune infiltration, prognosis, YKT6

## Abstract

**Background:**

YKT6 is markedly overexpressed across multiple tumor types and plays a role in driving their progression. However, the correlation between YKT6 and breast cancer remains poorly understood. Therefore, we aimed to investigate the potential prognostic value and biological function of YKT6 gene in breast cancer.

**Methods:**

Public datasets, clinic sample and tissue microarray (TMA) were used for YKT6 expression and prognostic value analyses. The relevance between YKT6 expression, tumor-immune infiltrates and tumor mutation burden (TMB) was examined using the TCGA database. Cellular functional assays were performed to verify the biological behavior of YKT6 in breast cancer cells. Moreover, transcriptome sequencing (RNA-seq) was conducted to explore the underlying mechanism of YKT6.

**Results:**

YKT6 was significantly upregulated in breast cancer tissue comparing to normal tissue(P<0.05) and higher YKT6 expression was significantly linked to worse clinical prognosis, advanced tumor stages, and distant metastasis(P<0.05). Additionally, YKT6 expression is correlated with the infiltration of various immune cell and TMB. Knockdown of YKT6 impaired the proliferation, invasion, and migration abilities of breast cancer cells, and increased apoptosis. Functional enrichment analysis revealed that YKT6 primarily influenced breast cancer progression through the cell cycle, as well as biological processes such as autophagy, apoptosis, and ferroptosis. Moreover, knockdown of YKT6 suppressed the activity of mTORC1.

**Conclusion:**

YKT6 may serve as a potential prognostic biomarker for breast cancer. The expression level of YKT6 was correlated with tumor-infiltrating immune cells in breast cancer. It may offer potential value for the treatment of breast cancer patients.

## 1 Introduction

Breast cancer remains the most common malignant tumor among women worldwide and is one of the leading causes of cancer-related mortality (1), posing a significant threat to women’s health. Despite significant advances in early detection and therapeutic strategies, the global burden of breast cancer continues to rise. Multiple risk factors contribute to breast cancer initiation and progression, including genetic susceptibility (such as BRCA1/2 mutations), hormonal exposure, lifestyle behaviors, and environmental influences. For individuals at high risk, regular screening and genetic counseling, maintenance of a healthy body weight, increased physical activity, limitation of alcohol consumption, and appropriate modulation of hormonal exposure are considered effective approaches to reduce breast cancer incidence (2, 3). In recent years, due to advancements in early diagnosis and precision therapy, early-stage breast cancer is considered curable, whereas the survival rate for advanced-stage patients remains at only 20%-30% (4). Breast cancer is a highly heterogeneous disease, and this complexity exists not only between patients but also within individual tumors. Such heterogeneity leads to markedly different clinical courses and therapeutic outcomes even among patients with apparently similar clinicopathological features. Consequently, there is still an urgent need to identify novel biomarkers to support personalized and precision management of breast cancer.

YKT6 is a member of the SNARE protein family and an R-SNARE. Its expression is elevated in several types of cancer. SNARE proteins drive membrane fusion processes (5). YKT6 has been found to be essential for the fusion of secretory vesicles with the plasma membrane (6). Studies have shown that disruption of YKT6 can inhibit the secretion of extracellular vesicles (7). In pancreatic cancer, YKT6 has been shown to promote the release of exosomes (8). The increase in hepatic exosome secretion may also be mediated through the YKT6 pathway (9). Additionally, YKT6 mediates the fusion of autophagosomes with lysosomes, and the fusion is inhibited when YKT6 is absent (10). The malfunction of these processes is linked to the pathogenesis of various human diseases, including cancer (11–13). Studies have shown that YKT6 is linked to the invasive phenotype and resistance mechanisms in breast cancer (14, 15). However, the role of YKT6 in breast cancer remains poorly understood and warrants further exploration and clarification.

## 2 Materials and methods

### 2.1 Data acquisition

RNA-seq data and clinical information for breast cancer cases were downloaded from The Cancer Genome Atlas (TCGA) database (https://portal.gdc.cancer.gov/), including 1,081 tumor tissues and 99 paired normal tissues. Gene expression levels were quantified as FPKM, followed by log2(FPKM + 1) transformation. Data visualization was performed using the ggplot2 package in R.

### 2.2 Collection of tumor and adjacent normal tissues

A total of 22 paired breast tumor tissues and adjacent non-tumor samples were collected from Fudan University Obstetrics and Gynecology Hospital. Written informed consent was obtained from all patients before sample collection, and the study was approved by the Ethics Committee of Fudan University Obstetrics and Gynecology Hospital. All procedures were conducted in accordance with the principles of the Declaration of Helsinki.

### 2.3 RNA extraction and RT-qPCR

Total RNA was extracted from cells or tissues using the EZB RNA Extraction Kit (EZBioscience, USA) according to the manufacturer’s protocol. For cDNA synthesis, 1 µg of total RNA was reverse-transcribed using the color Reverse Transcription Kit (EZBioscience, USA) in a 20 µL reaction volume following the manufacturer’s instructions. The resulting cDNA was diluted 1:10 with nuclease-free water and used for quantitative PCR. RT-qPCR was performed using a QuantStudio 6 Flex Real-Time PCR System (Applied Biosystems, USA) with the SYBR Green qPCR Master Mix (EZBioscience, USA). Gene-specific primers were designed using Primer-BLAST (NCBI) and synthesized by Tsingke Biotechnology Co., Ltd (Beijing, China). Relative gene expression levels were calculated using the 2^−ΔΔCt method, with ACTIN serving as the internal reference gene.

### 2.4 Immunohistochemistry

Human breast cancer tissue microarrays (TMAs) (HBreD67SU02) were provided by Shanghai Outdo Biotech Co., Ltd. The TMAs were deparaffinized in xylene and hydrated using a graded series of ethanol concentrations followed by distilled water. Antigen retrieval was carried out using EDTA antigen retrieval buffer. Endogenous peroxidase activity was inhibited using a 3% hydrogen peroxide solution. The tissue sections were blocked with 3% BSA (Solarbio, China). The sections were incubated overnight at 4 °C with anti-YKT6 antibody (Thermo Fisher Scientific, #PA556565, 1:10). After washing, the sections were incubated at room temperature with secondary antibody (Abcam, #ab205718, 1:5000) for 50 minutes. After applying DAB chromogen (DAKO, Denmark), the color development time was controlled under a microscope. The sections were counterstained with hematoxylin for 3 minutes, washed with running water, dehydrated, and mounted.

### 2.5 Analysis of immune cell infiltration in tumor microenvironment

Immune cell infiltration in breast cancer tumor samples from the TCGA database was analyzed using the CIBERSORT and ssGSEA methods in R. The differences in immune cell infiltration between the high and low YKT6 expression groups were analyzed using the Wilcoxon rank-sum test. The correlation between YKT6 expression and the levels of immune cell infiltration was assessed using Spearman’s rank correlation analysis.

### 2.6 Tumor mutation burden and drug sensitivity

Somatic mutation data were obtained from the TCGA database, and the “maftools” package was used to construct the somatic mutation landscape of YKT6 expression in breast cancer. In addition, data from the Genomics of Drug Sensitivity in Cancer (GDSC: https://www.cancerrxgene.org/) database were used as the training set, while TCGA data were used as the validation set to evaluate the impact of YKT6 expression levels on the chemotherapeutic drug sensitivity of breast cancer patients.

### 2.7 RNA-seq and functional enrichment analysis

Total RNA was extracted from si-YKT6 or si-NC-transfected MCF-7 cells (n = 3 per group) and sequenced on an Illumina Novaseq 6000 platform to generate 150 bp paired-end reads. Raw reads were processed using fastp for quality control, and low-quality reads were removed. Clean reads were aligned to the reference genome using HISAT2, and gene expression levels were quantified as FPKM. Raw read counts were obtained using HTSeq-count. Differential expression analysis was performed using the DESeq2 R package. To control false positives, P values were adjusted using the Benjamini–Hochberg (BH) procedure (FDR < 0.05) and |log2FC| > 0.58 as the threshold for significantly differentially expressed genes (DEGs). Similarly, for GO, KEGG and GSEA enrichment analyses, the BH correction was applied, with an adjusted p-value (FDR) < 0.05 considered statistically significant.

### 2.8 Antibodies and western blot

Protein samples were prepared on ice using RIPA lysis buffer (NCM Biotech, China) supplemented with a protease phosphatase inhibitor (NCM Biotech, China). Protein concentrations were determined using the BCA protein assay kit (Epizyme, China). Subsequently, proteins were separated by SDS-PAGE and transferred to a PVDF membrane (Bio-Rad, USA). The membrane was blocked with blocking buffer (Servicebio, USA) for 15 minutes at room temperature, followed by overnight incubation with primary antibodies at 4 °C. After washing with TBST, the membrane was incubated with secondary antibodies for 2 hours at room temperature. Antibody signals were detected using the ECL detection kit (NCM Biotech, China). Rabbit Anti-Human YKT6 Polyclonal Antibody (Thermo Fisher Scientific, #PA556565, 1:1000), Rabbit Anti-Human ACTIN Polyclonal Antibody (Servicebio, #GB15003,1:2000), Rabbit Anti-Human Akt Polyclonal Antibody (Starter Biotechnology, #S0B0114, 1:500), Rabbit Anti-Human p-Akt Polyclonal Antibody (Ser473) (CST,1:1000, #9271), Mouse Anti-Human mTOR Polyclonal Antibody (Proteintech, #66888-1, 1:1000), Rabbit Anti-Human p-mTOR Polyclonal Antibody(Ser2448) (CST, #5536T, 1:1000).

### 2.9 Cell culture and cell transfection

Human mammary epithelial cell lines (MCF-10A) were purchased from Shanghai QuiCell Biotechnology Co., Ltd. & Technology Co., Ltd. All breast cancer cell lines (MDA-MB-231, MDA-MB-468, BT-549, MCF-7) were obtained from the Shanghai Cell Bank of the Chinese Academy of Sciences (Shanghai, China). MCF-10A cell lines were cultured in MCF-10A-specific medium (QuiCell, China), MDA-MB-231, MDA-MB-468, MCF-7cell lines were cultured in DMEM (BasalMedia, China), BT-549 cells were cultured in RPMI-1640(BasalMedia, China). All culture media were supplemented with 10% fetal bovine serum (VivaCell, China) and 1% penicillin-streptomycin (NCM Biotech, China). All cells were at 37 °C in a 5% CO2 atmosphere.

For transient transfection, MDA-MB-231 and MCF-7cells were seeded in 6-well plates. Cells transfected with CALNPTMRNAi *in vitro* (Duona, China). After 24 hours, the medium was replaced with fresh medium containing 10% fetal bovine serum, and incubation was continued for subsequent experiments. YKT6 specific small interfering RNA (siYKT6) and non-specific control siRNA (si-NC) were purchased from GenePharma (Shanghai, China). Their target sequences are as follows:

si-YKT6-sense CCAGCGUUCAGGAAUUCAUTT
si-YKT6-antisense AUGAAUUCCUGAACGCUGGTT
si-NC-sense UUCUCCGAACGUGUCACGUTT
si-NC-antisense ACGUGACACGUUCGGAGAATT

### 2.10 Scratch wound healing assay

Cells were seeded into 6-well plates at a density of 1 × 10^5^ cells per well. Once the cells reached approximately 90% confluence, a sterile 20 µL pipette tip was used to create a vertical scratch perpendicular to the horizontal axis. After washing with PBS, the cells were cultured in serum-free medium, and images were captured at 0, 48hours post-scratch using a phase-contrast microscope (Olympus, Japan). The migration rate was quantified by measuring the wound closure distance using ImageJ software.

### 2.11 Transwell assay

Cells treated with siRNA were cultured in serum-free medium for 24 hours. The upper surface of the transwell inserts with an 8 µm pore size (Corning, USA) was pre-coated with Matrigel (Corning, USA), and the cell density was adjusted to 2 × 10^5^ cells/mL in serum-free medium. A 200 µL cell suspension was added to the upper chamber, while 600 µL complete medium containing 10% FBS was added to the lower chamber. The cells were then incubated for 48 hours in a cell culture incubator. The cells that had migrated to the underside of the membrane were fixed with formaldehyde and stained with crystal violet. The stained cells were photographed under a light microscope (Olympus, Japan), and the number of invasive cells was counted.

### 2.12 Flow-cytometry analysis

Cell apoptosis was evaluated using the Annexin V-APC/PI Apoptosis Detection Kit (BioLegend, USA) according to the manufacturer’s instructions. Briefly, Cells 48 hours post-siRNA transfection were harvested by trypsinization, washed with cold PBS, and resuspended in Annexin V Binding Buffer. Subsequently, Annexin V-APC and PI were added to the cell suspension, and the mixture was incubated at room temperature in the dark for 15 minutes. After incubation, an appropriate volume of Annexin V Binding Buffer was added. The samples were immediately analyzed using a flow cytometer (Beckman Coulter, USA) and analyzed using FlowJo software (Tree Star, USA).

The cell cycle assay was performed according to the protocol using a cell cycle staining kit (LiankeBio, China). After propidium iodide staining, the samples were analyzed by a flow cytometer (Beckman Coulter, USA), and the percentages of cells in G1, S, and G2-M phases were counted and compared.

### 2.13 CCK-8 assay

Cell proliferation was evaluated using the Cell Counting Kit-8 (CCK-8) (NCM Biotech, China). Cells were seeded into 96-well plates at a number of 1 × 10^3^ cells per well. After 24, 48, 72 and 96 hours, 10 µL of CCK-8 reagent was added to each well, and cells were incubated for an additional 2 hours. The absorbance at 450 nm was measured using a microplate reader (BioTek, USA) to assess cell viability. Cell proliferation curves were analyzed using two-way ANOVA, and the interaction effect between time and treatment was used to assess differences between groups.

### 2.14 Colony formation assay

Cells were plated into six-well plates at a defined density and maintained at 37 °C in a 5% CO_2_ incubator for 10–14 days, with fresh medium changed every two days. The cells were then fixed in 4% paraformaldehyde for 30 minutes, stained with crystal violet for 30 minutes, imaged, and the colony numbers were quantified for statistical analysis.

### 2.15 Statistical analysis

All analyses were conducted using R (v4.2.0 for TCGA; v3.2.0 for RNA-seq) and GraphPad Prism (v10.0). For TCGA data, group comparisons were performed using the Wilcoxon rank-sum test, and paired samples were analyzed using the Wilcoxon signed-rank test. Non-parametric methods were used to accommodate non-normal distributions and unequal sample sizes. Survival outcomes were analyzed using the Kaplan-Meier method with the log-rank test. Univariate and multivariate Cox proportional hazards models were used to identify independent prognostic factors. For analyses involving multiple comparisons, P values were adjusted using the Benjamini–Hochberg procedure to control the false discovery rate (FDR). Associations between YKT6 expression and immune cell infiltration were analyzed using Spearman’s rank correlation coefficient. For *in vitro* assays, data normality was assessed using the Shapiro–Wilk test. Two-tailed Student’s t-test or non-parametric tests were applied as appropriate. Two-way ANOVA was used for CCK-8 assays. A two-sided P < 0.05 (or FDR < 0.05) was considered significant. All experimental data are presented as mean ± SD from at least three independent biological replicates.

## 3 Results

### 3.1 Elevated expression of YKT6 in breast cancer tissues

**Figure 1:**
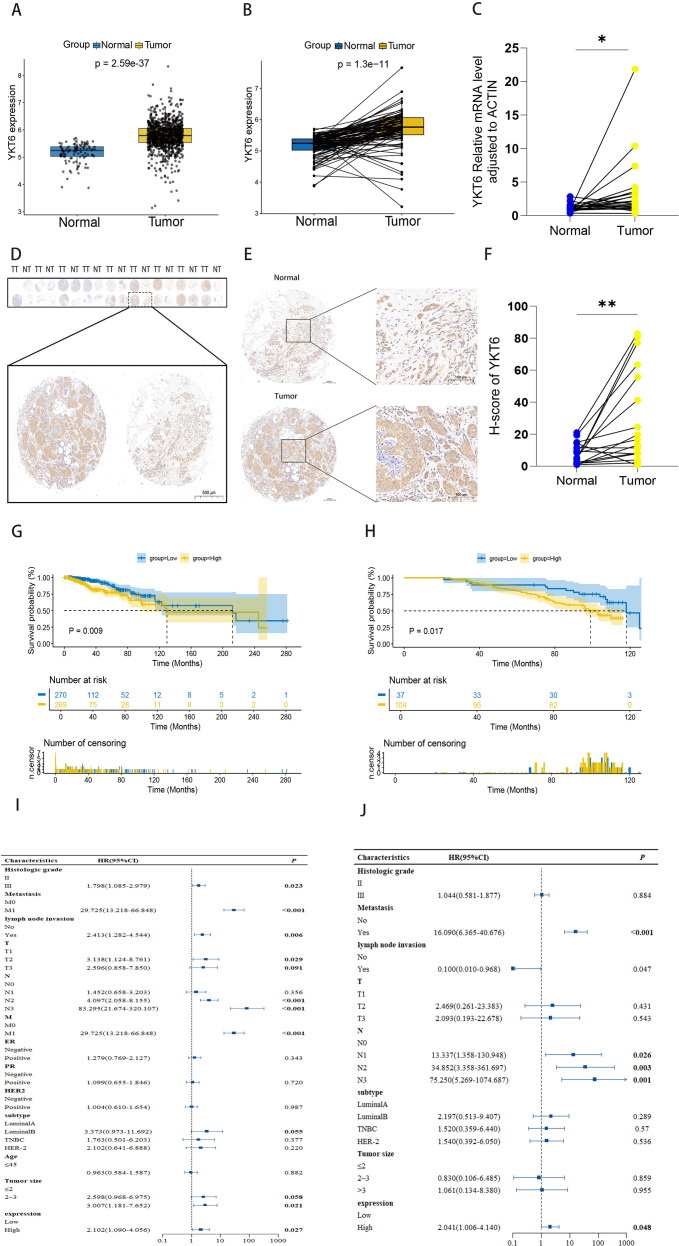
YKT6 expression is associated with poor prognosis in patients with breast cancer: **(A)** Expression of YKT6 mRNA in unpaired breast tumor tissues (n=1,081) and normal tissues (n=99) from the TCGA database. **(B)** Expression of YKT6 mRNA in paired breast tumor tissues and adjacent normal tissues (n=98) from the TCGA database. **(C)** Expression of GENE in paired breast tumor tissues and adjacent normal tissues (n=22) from clinical samples. **(D)** Tissue microarray (TMA) analysis of YKT6 expression in normal and breast tumor tissues. **(E)** Representative immunohistochemical (IHC) staining of YKT6 in normal and breast tumor tissues. **(F)** H-score analysis of YKT6 staining in normal and breast tumor tissues (n=17) form TMA. **(G)** Association of YKT6 expression with Overall Survival (OS) in breast cancer based on the TCGA database. **(H)** Association of YKT6 expression with Overall Survival (OS) in breast cancer validated by TMA. **(I)** Univariate Cox proportional hazards regression analysis evaluating the prognostic value of YKT6 and clinicopathological variables. **(J)** Multivariate Cox proportional hazards regression analysis to determine whether YKT6 is an independent prognostic factor. For comparisons between two independent groups, the Wilcoxon rank-sum test was applied. For paired samples, the Wilcoxon signed-rank test was used. Survival curves were generated using the Kaplan–Meier method, and survival differences were compared using the log-rank test. Univariate and multivariate Cox proportional hazards regression analyses were performed to evaluate the independent prognostic value of YKT6 expression, with hazard ratios (HRs) and 95% confidence intervals (CIs) reported. P values were two-sided. *P < 0.05, **P < 0.01.

Firstly, we obtained unpaired and paired BRCA data from the TCGA database (Tumor, n = 1081; Normal, n = 99). Both paired and unpaired analyses revealed that YKT6 mRNA expression was significantly upregulated in tumor tissues compared to normal tissues (P < 0.001) (**Figures 1A, B**). To further validate its expression in breast cancer, we collected 22 pairs of breast tumor tissues and matched adjacent normal tissues for qRT-PCR analysis. The results confirmed that YKT6 mRNA expression levels were significantly higher in breast cancer tissues than in matched normal tissues (P < 0.05) (**Figure 1C**). Furthermore, we confirmed through immunohistochemistry (IHC) that the expression of YKT6 protein in breast tumor tissues was significantly higher compared to normal tissues. (P < 0.01) (**Figures 1D–F**).

### 3.2 The expression of YKT6 is associated with poor prognosis in breast cancer patients

To further elucidate the relationship between YKT6 expression and prognosis, we utilized the KM plotter database (www.kmplot.com) to investigate the association between YKT6 expression and patient prognosis. As shown in the figure (**Supplementary Figure 1A**), YKT6 expression was not significantly correlated with overall survival (OS) in breast cancer patients (P > 0.05), but it was significantly associated with relapse-free survival (RFS) (P < 0.001) (**Supplementary Figure 1B**). Subsequently, we analyzed TCGA data and classified the gene expression into high and low expression groups based on quartiles. Survival curves were generated using the survival and survminer packages, which indicated that patients with high YKT6 expression had shorter OS (**Figure 1G**). Additionally, using data from tissue microarrays (TMA), YKT6 expression was divided into high and low expression groups based on the optimal cutoff value, and survival curves were plotted, yielding consistent results (**Figure 1H**). These data suggest that high YKT6 expression is associated with worse prognosis in breast cancer patients.

Subsequently, univariate and multivariate Cox analyses were performed using data from tissue microarrays (TMA), and forest plots were generated (**Figures 1I, J**). The results indicated that YKT6 expression can serve as an independent prognostic factor for breast cancer patients. The baseline characteristics of the TMA data are summarized in **Table 1**.

**Table 1:** The baseline characteristics of the TMA data.

Variables	Category	YKT6 low expression	YKT6 high expression	p
n		37	104	
Histologic grade (%)	II	25 (67.6)	55 (52.9)	0.175
	III	12 (32.4)	49 (47.1)	
Metastasis (%)	No	33 (89.2)	90 (86.5)	0.898
	Yes	4 (10.8)	14 (13.5)	
lymph node invasion (%)	No	11 (29.7)	35 (33.7)	0.816
	Yes	26 (70.3)	69 (66.3)	
status (%)	Alive	23 (62.2)	49 (47.1)	0.167
	Dead	14 (37.8)	55 (52.9)	
T (%)	T1	3 (8.1)	17 (16.3)	0.3
	T2	23 (62.2)	66 (63.5)	
	T3	11 (29.7)	21 (20.2)	
N (%)	N0	11 (29.7)	34 (32.7)	0.933
	N1	13 (35.1)	31 (29.8)	
	N2	12 (32.4)	35 (33.7)	
	N3	1 (2.7)	4 (3.8)	
M (%)	M0	33 (89.2)	90 (86.5)	0.898
	M1	4 (10.8)	14 (13.5)	
TNM stage (%)	I	2 (5.4)	10 (9.6)	0.818
	II	17 (45.9)	42 (40.4)	
	III	14 (37.8)	38 (36.5)	
	IV	4 (10.8)	14 (13.5)	
ER (%)	Negative	20 (54.1)	62 (59.6)	0.812
	Positive	15 (40.5)	36 (34.6)	
	Unknown	2 (5.4)	6 (5.8)	
PR (%)	Negative	18 (48.6)	65 (62.5)	0.294
	Positive	17 (45.9)	33 (31.7)	
	Unknown	2 (5.4)	6 (5.8)	
HER2 (%)	Negative	18 (48.6)	49 (47.1)	0.986
	Positive	17 (45.9)	49 (47.1)	
	Unknown	2 (5.4)	6 (5.8)	
Subtype (%)	LuminalA	4 (10.8)	7 (6.7)	0.605
	LuminalB	4 (10.8)	21 (20.2)	
	TNBC	10 (27.0)	21 (20.2)	
	HER-2	17 (45.9)	47 (45.2)	
	Unknown	2 (5.4)	8 (7.7)	
Age(%)	≤45	15 (40.5)	43 (41.3)	1
	>45	22 (59.5)	61 (58.7)	
Tumor size (%)	≤2	5 (13.5)	19 (18.3)	0.803
	2~3	11 (29.7)	29 (27.9)	
	>3	21 (56.8)	56 (53.8)	

### 3.3 Correlation of YKT6 gene expression with clinicopathological characteristics

**Figure 2:**
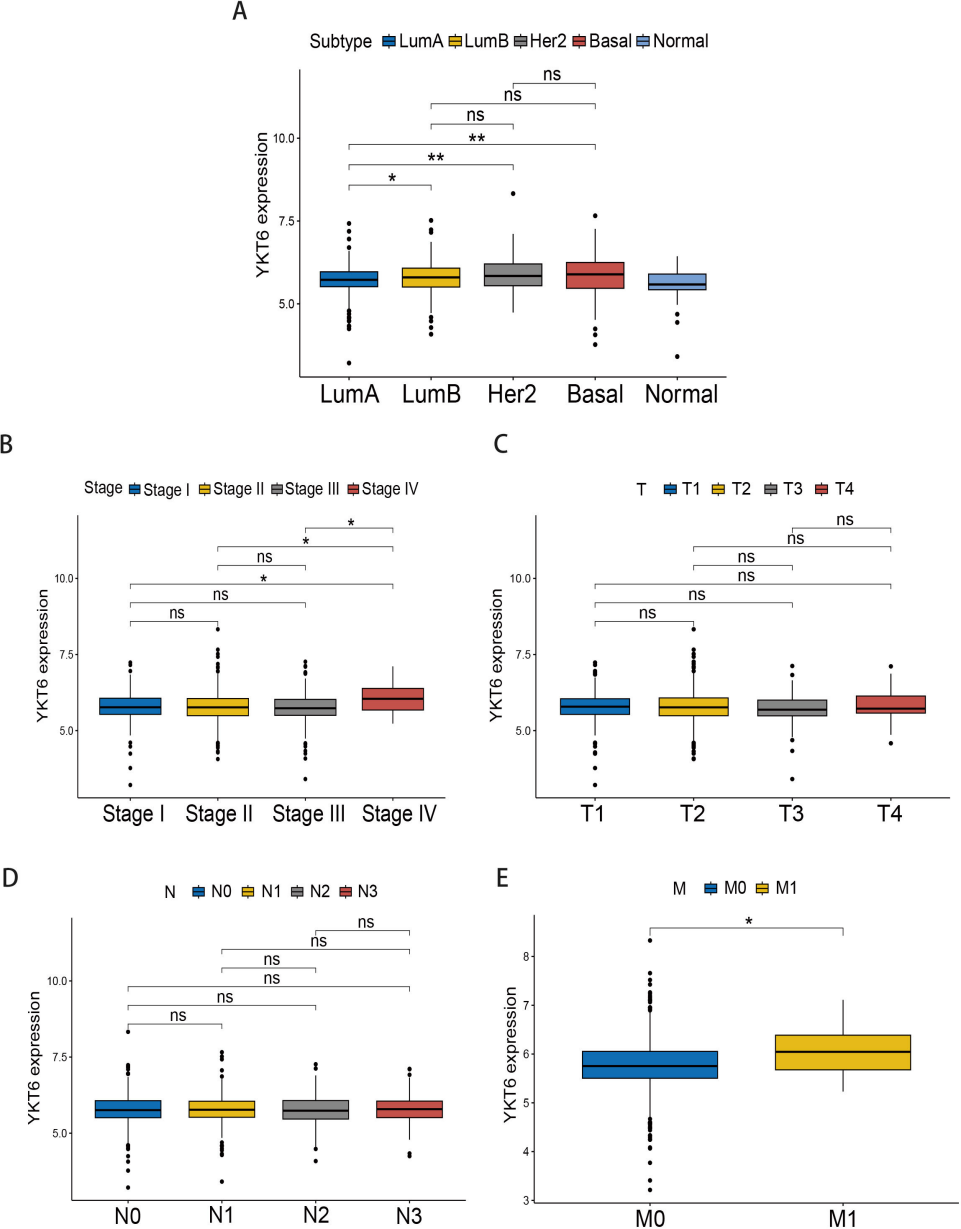
Correlation of YKT6 gene expression with clinicopathological characteristics: **(A)** the expression of YKT6 in different breast cancer subtypes, **(B)** expression of YKT6 across different pathological stages, **(C)** expression of YKT6 across different T stages, **(D)** expression of YKT6 across different N stages, **(E)** expression of YKT6 across different M stages. Wilcoxon rank-sum test was used for pairwise comparisons. P values were two-sided. Data are presented as boxplots showing the median and interquartile range (IQR). *P < 0.05, **P < 0.01, ns, no significant difference.

We analyzed the correlation between YKT6 expression and clinical features using data from the TCGA database. The results indicated that, although YKT6 expression was not associated with T or N stage (P>0.05) (**Figures 2C, D**), its expression was significantly higher in metastatic patients (M1) compared to non-metastatic patients (M0) (P<0.05) (**Figure 2E**). Meanwhile YKT6 expression was elevated in advanced-stage patients (stage IV) compared to patients in earlier stages and locally advanced (stage I, stage II, and stage III) (P<0.05) (**Figure 2B**). Furthermore, YKT6 expression was higher in more aggressive breast cancer subtypes (Luminal B, HER2, Basal) compared to the better-prognosis subtype (Luminal A) (P<0.05) (**Figure 2A**).

### 3.4 Immune infiltration profile in relation to YKT6 expression in breast cancer

**Figure 3:**
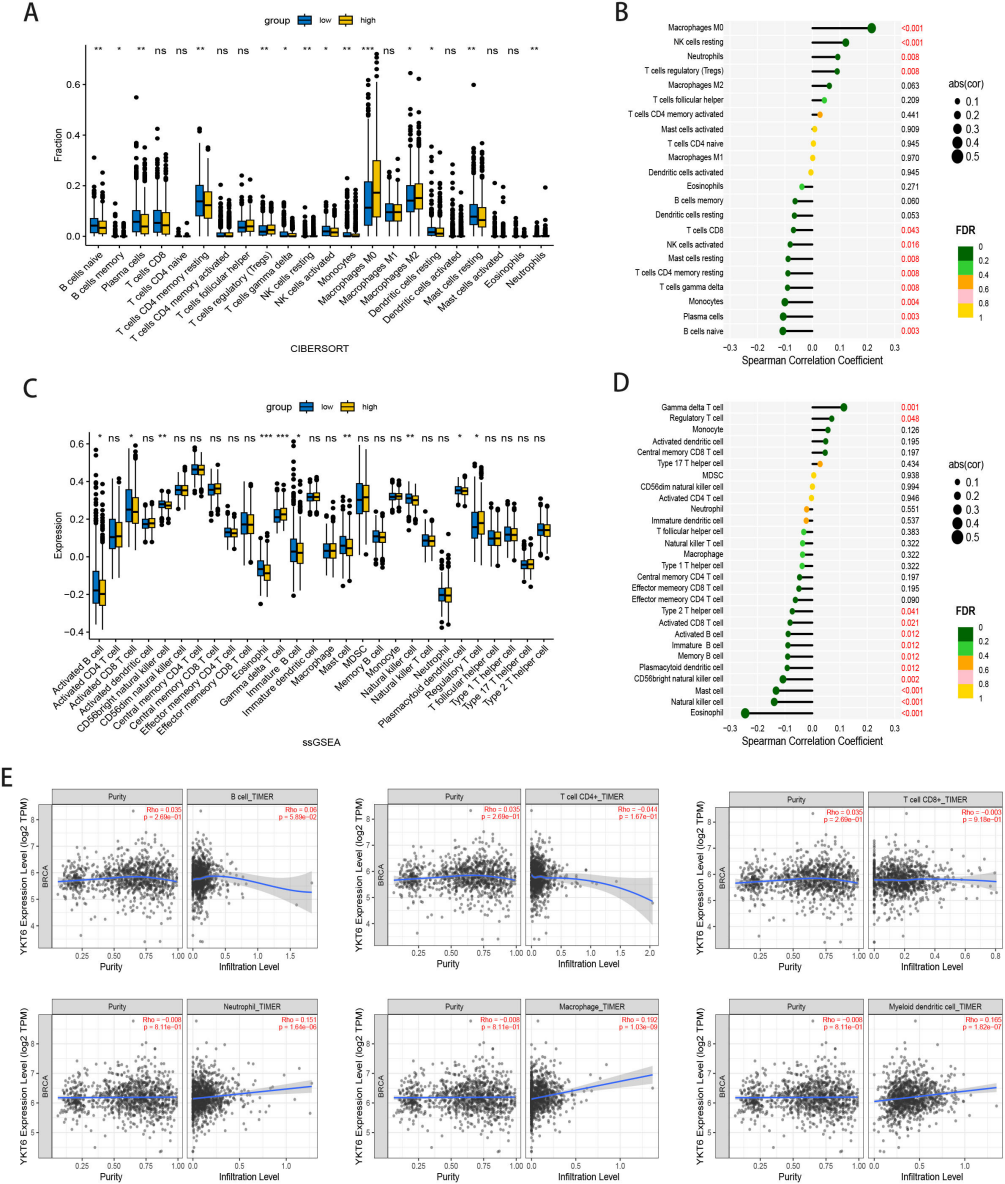
Immune infiltration profile in relation to YKT6 expression in breast cancer: **(A)** Differences in immune cell expression between high and low YKT6 expression breast cancer patients: CIBERSORT. **(B)** Correlation analysis between YKT6 expression and immune cell infiltration presented as lollipop plots: CIBERSORT. **(C)** Differences in immune cell expression between high and low YKT6 expression breast cancer patients: ssGSEA. **(D)** Correlation analysis between YKT6 expression and immune cell infiltration presented as lollipop plots: ssGSEA. **(E)** Correlation Between YKT6 Expression and Immune Cell Infiltration Based on TIMER Algorithm. Statistical comparisons were performed using the Wilcoxon rank-sum test. Correlations were assessed using Spearman’s rank correlation analysis. *P < 0.05, **P < 0.01, ***P < 0.001, ns, no significant difference.

We analyzed the differences in immune cell infiltration between high and low YKT6 expression patients using TCGA data and assessed the correlation between YKT6 expression and immune cell infiltration using Spearman’s correlation test. CIBERSORT analysis showed that YKT6 expression was associated with higher proportions of regulatory T cells and lower proportions of several immune cell populations, including CD8+ T cells, γδ T cells, activated NK cells, and plasma cells (**Figure 3B**). Moreover, using the TIMER 2.0 database and TIMER algorithm, we assessed the association between YKT6 and various immune cell types. The results showed that YKT6 expression was positively correlated with neutrophils, macrophages, and myeloid dendritic cells(Rho = 0.151, p = 1.64e – 06; Rho = 0.192, p=1.03e-09; Rho = 0.165, p=1.82e-07), but not with other immune cell populations (**Figure 3E**). To address the potential influence of outliers, a sensitivity analysis was performed by excluding extreme values (top and bottom 1%) in TCGA data. After exclusion, the association between YKT6 and neutrophils was no longer significant (**Supplementary Figure 2**). Overall, similar trends were observed across TIMER, CIBERSORT, and ssGSEA analyses, although the strength of the associations was modest and appeared to be influenced by potential outliers. These findings suggest that YKT6 may be involved in shaping the tumor immune microenvironment, although further studies are required to clarify its functional role. Subsequently, we assessed the relationship between YKT6 expression and eight inhibitory immune checkpoints. The analysis revealed that patients with high YKT6 expression exhibited significant upregulation of HAVCR2 (TIM-3) (P<0.001). No significant correlations were found with other (**Supplementary Figure 3**).

### 3.5 Potential role of YKT6 in genomic alterations in breast cancer

**Figure 4:**
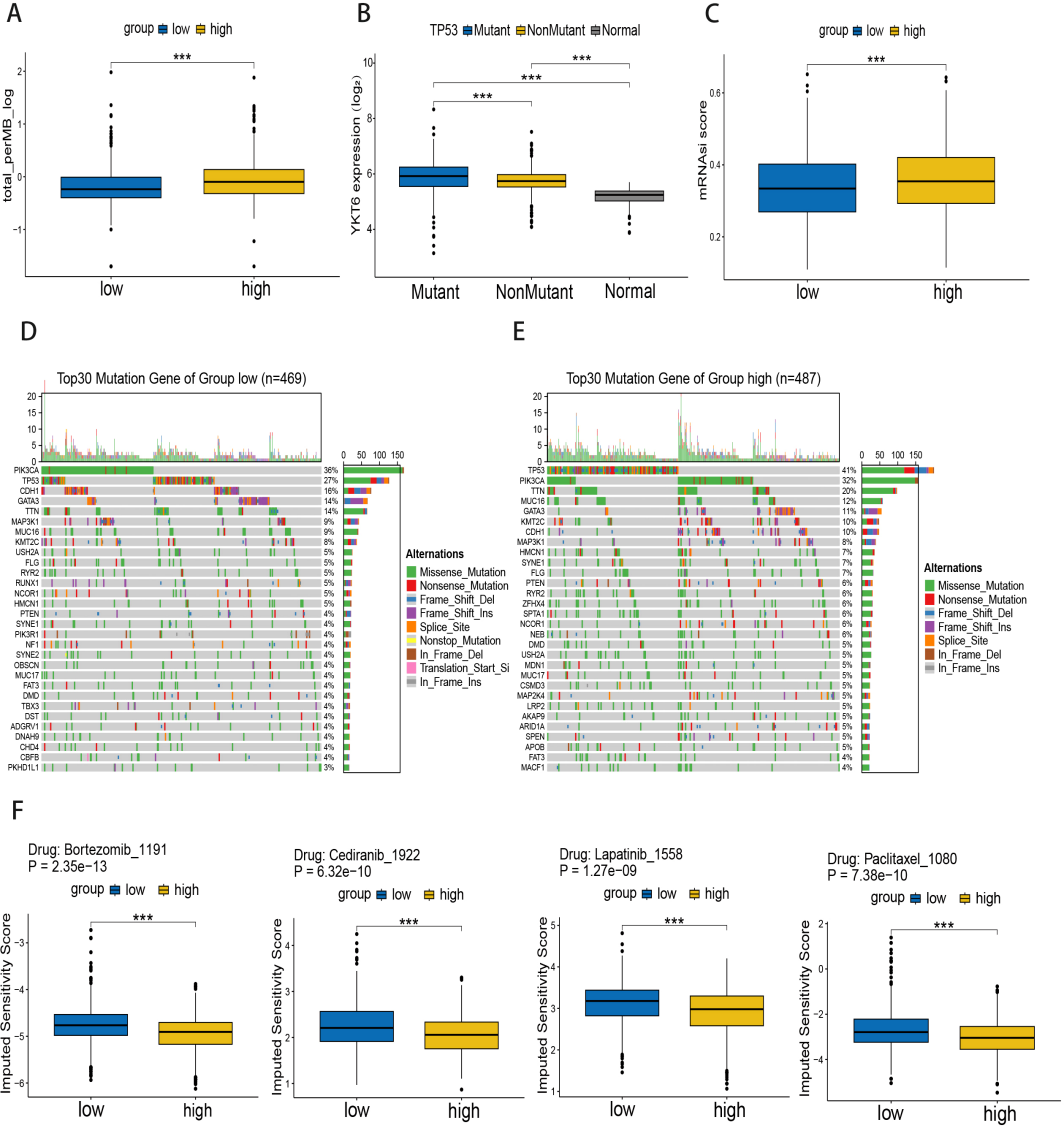
Potential role of YKT6 in genomic alterations in breast cancer: **(A)** Comparison of tumor mutation burden (TMB) between high and low YKT6 expression groups. **(B)** Comparison of YKT6 expression among TP53-mutant tumors, TP53-wildtype tumors, and normal breast tissues in the TCGA-BRCA cohort. **(C)** Comparison of mRNA expression-based stemness index (mRNAsi) scores between high and low YKT6 expression groups. **(D, E)** Somatic mutation landscapes of breast cancer patients with low **(D)** and high **(E)** YKT6 expression. The top 30 most frequently mutated genes are shown. **(F)** Predicted drug sensitivity analysis comparing estimated half-maximal inhibitory concentration (IC50) values between high and low YKT6 expression groups. Data are presented as boxplots showing median and interquartile range. Statistical comparisons between groups were performed using the Wilcoxon rank-sum test. ***P < 0.001.

To investigate the relationship between gene mutations and YKT6 expression levels, we downloaded somatic mutation data of breast cancer from the TCGA database and analyzed the tumor mutational burden between high and low YKT6 expression groups. The results showed that the tumor mutational burden was significantly increased in the high YKT6 expression group (**Figure 4A**), suggesting that YKT6 expression levels might be associated with genomic instability and mutation rate. We subsequently generated waterfall plots for the low and high YKT6 expression groups (**Figures 4D, E**), which revealed a significantly higher frequency of TP53 mutations in the high YKT6 group (**Figure 4B**), consistent with a previous study of YKT6 in oral squamous cell carcinoma (16). We note that this finding represents a statistical association within the cohort and does not establish whether YKT6 overexpression is a driver or a consequence of oncogenic transformation. We then investigated the relationship between YKT6 expression levels and mRNAsi scores, and found that patients with high YKT6 expression had higher mRNAsi scores (**Figure 4C**). Furthermore, to evaluate the impact of YKT6 expression on the chemotherapeutic drug sensitivity of breast cancer patients, we conducted drug sensitivity scoring. We found that YKT6 expression was predominantly negatively correlated with bortezomib, cediranib, paclitaxel, and lapatinib, which may provide potential reference value for or future clinical therapies specifically targeting YKT6 (**Figure 4F**).

### 3.6 YKT6 knockdown reduces wound closure in breast cancer cells

**Figure 5:**
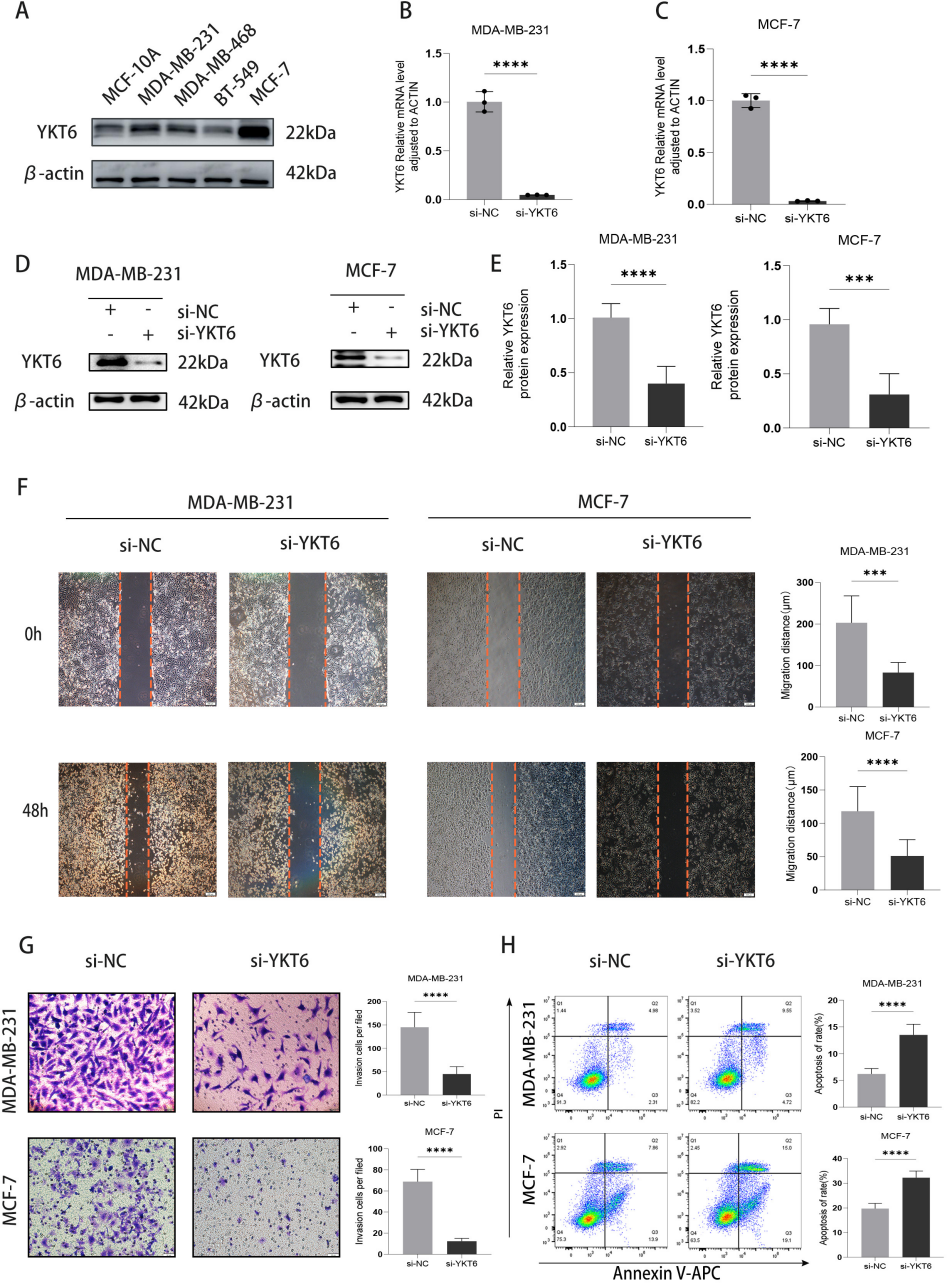
YKT6 knockdown inhibits migration in breast cancer cells: **(A)** Expression of YKT6 in five different breast cancer cell lines. **(B)** Validation of YKT6 knockdown in MCF-7 cells by qPCR. **(C)** Validation of YKT6 knockdown in MDA-MB-231 cells by qPCR. **(D, E)** western blot were used to verify the efficiency of YKT6 knockdown. **(F)** Cell migratory capacity was assessed using a wound-healing (scratch) assay in MCF-7 and MDA-MB-231 cells transfected with si-NC or si-YKT6. **(G)** Cell invasion ability was evaluated using a Transwell invasion assay in MDA-MB-231 and MCF-7 cells transfected with si-NC or si-YKT6. **(H)** Apoptosis was analyzed by flow cytometry using Annexin V–APC/PI staining in MDA-MB-231 and MCF-7 cells transfected with si-NC or si-YKT6. All experiments were independently repeated at least three times (n = 3 biological replicates). Data are presented as mean ± standard deviation (SD). Statistical comparisons between si-NC and si-YKT6 groups were performed using two-tailed Student’s t-test. ***p < 0.001,****p < 0.0001. The uncropped original blots corresponding to this figure are provided in **Supplementary Figure 5**.

To evaluate the biological functions of YKT6 in breast cancer, we first examined its expression across a panel of breast epithelial and breast cancer cell lines. YKT6 expression was detected in all tested cell lines, with comparatively higher levels observed in MCF-7 and MDA-MB-231 cells relative to the non-tumorigenic MCF-10A cells (**Figure 5A**). These two cell lines were subsequently selected for YKT6 knockdown to evaluate its functional impact. As shown in **Figures 5B–E**, YKT6 mRNA and protein was successfully silenced in MCF-7 and MDA-MB-231 cells (P<0.001).Wound-healing assays showed that YKT6 knockdown significantly reduced wound closure in both MCF-7 and MDA-MB-231 cells (**Figure 5F**, P < 0.001). In MDA-MB-231 cells, this reduction was evident at 24 hours post-scratch (**Supplementary Figure 4**), prior to detectable proliferation differences. In contrast, significant differences in MCF-7 cells were observed at 48 hours, at which time proliferation effects may also contribute to the phenotype.

### 3.7 YKT6 knockdown inhibits invasion, and promoting apoptosis in breast cancer cells

Transwell assays were performed to assess the invasive capacity of the cells. The results showed a substantial reduction in invasion following YKT6 knockdown (P<0.001) (**Figure 5G**). Moreover, silencing YKT6 led to a significant increase in cell apoptosis (P<0.001) (**Figure 5H**).

### 3.8 YKT6 knockdown inhibits proliferation in breast cancer cells

**Figure 6:**
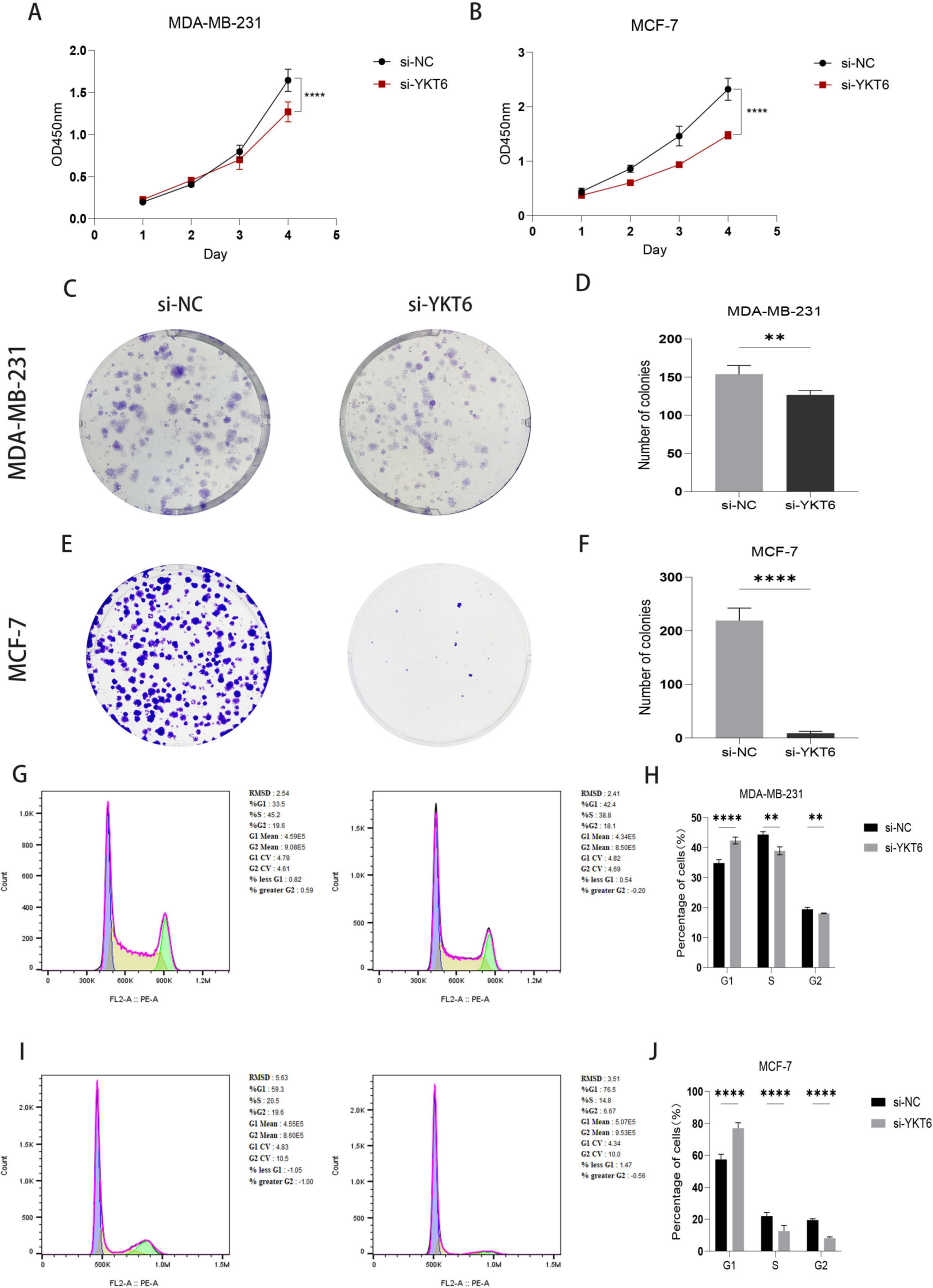
YKT6 knockdown inhibits proliferation in breast cancer cells: **(A, B)** Cell viability was evaluated using CCK-8 assays in MDA-MB-231 and MCF-7 cells transfected with si-NC or si-YKT6. **(C–F)** Colony formation capacity was assessed in MDA-MB-231 and MCF-7 cells transfected with si-NC or si-YKT6. **(G–J)** Cell cycle distribution was analyzed by flow cytometry in MDA-MB-231 and MCF-7 cells transfected with si-NC or si-YKT6. All experiments were independently repeated at least three times (n = 3 biological replicates). Data are presented as mean ± SD. CCK-8 data were analyzed using two-way ANOVA, while other comparisons between si-NC and si-YKT6 groups were analyzed using two-tailed Student’s t-test. **p < 0.01, ****p < 0.0001.

To further assess the effect of YKT6 knockdown on breast cancer cell proliferation, CCK-8 and colony formation assays showed that cell proliferative capacity was significantly reduced following YKT6 knockdown (**Figures 6A–F**). Cell cycle analysis revealed that after YKT6 knockdown, the proportion of MCF-7 and MDA-MB-231 cells in G1/G0 phase increased significantly, while the proportions of S and G2 phases decreased, indicating a substantial reduction in breast cancer cell proliferation (**Figures 6G–J**).

### 3.9 RNA-seq analysis of YKT6-regulated molecular pathways

To further investigate the molecular pathways and biological processes regulated by YKT6, we performed transcriptome sequencing (RNA-seq) after YKT6 knockdown in MCF-7 cells. Using the thresholds of p-value < 0.05 and FC > 1.5, a total of 2464 differentially expressed genes (DEGs) were identified, including 1084 upregulated genes and 1380 downregulated genes. The expression profiles of the DEGs are depicted in **Supplementary Figures 6B, C**.

**Figure 7:**
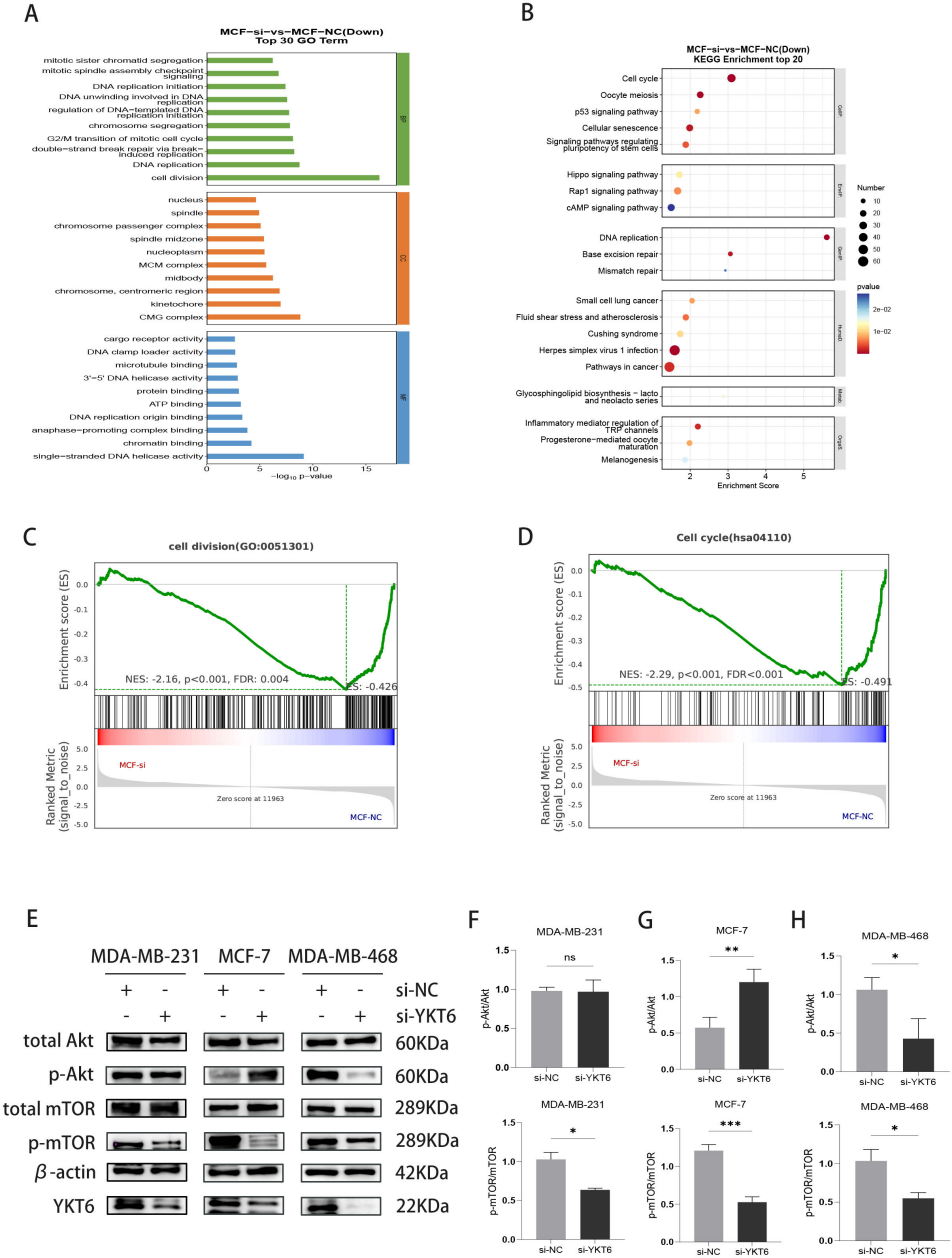
YKT6 knockdown is associated with suppression of cell cycle programs and modulation of PI3K–AKT–mTOR signaling: **(A)** Bar Plot of down-regulated gene GO enrichment after knockdown of YKT6 gene. **(B)** Bubble map of down-regulated gene KEGG enrichment after knockdown of YKT6 gene. **(C)** GSEA reveals suppression of cell division programs after YKT6 knockdown **(D)** GSEA reveals inhibition of cell cycle progression after YKT6 knockdown **(E)** Western blot analysis of YKT6, total AKT, phospho-AKT (Ser473), total mTOR, and phospho-mTOR (Ser2448) in MDA-MB-231, MCF-7, and MDA-MB-468 cells transfected with si-NC or si-YKT6. β-actin was used as a loading control. **(F)** Quantification of p-AKT (Ser473)/AKT and p-mTOR (Ser2448)/mTOR ratios in MDA-MB-231 cells transfected with si-NC or si-YKT6. **(G)** Quantification of p-AKT (Ser473)/AKT and p-mTOR (Ser2448)/mTOR ratios in MCF-7 cells transfected with si-NC or si-YKT6. **(H)** Quantification of p-AKT (Ser473)/AKT and p-mTOR (Ser2448)/mTOR ratios in MDA-MB-468 cells transfected with si-NC or si-YKT6. All experiments were independently repeated at least three times (n = 3 biological replicates per condition). Data are shown as mean ± standard deviation (SD). Statistical comparisons between si-NC and si-YKT6 groups were performed using a two-tailed Student’s t-test. *P < 0.05, **P < 0.01, ***P < 0.001, ns, no significant difference. The uncropped original blots corresponding to this figure are provided in **Supplementary Figures 7–9**.

Gene Ontology (GO) analysis was performed on the differentially expressed genes. The results showed that, after YKT6 knockdown, significant enrichment was observed in biological processes (BP) such as cell division, double-strand break repair via break-induced replication, G2/M transition of the mitotic cell cycle, and chromosome segregation (**Figure 7A**). Additionally, KEGG enrichment analysis revealed that downregulated genes were significantly enriched in several biological pathways, including the cell cycle, oocyte meiosis, p53 signaling pathway, and cellular senescence, with the cell cycle pathway being the most significantly enriched (**Figure 7B**).

In addition to GO and KEGG enrichment analyses, we also performed GESA analysis, which yielded consistent results. The analysis showed a significant downregulation of cell division (**Figure 7C**), DNA replication, chromosome segregation, G2/M transition of the mitotic cell cycle, and mitotic cell cycle (**Supplementary Figures 6D-F, J**). Conversely, autophagy, mitophagy, and ferroptosis were significantly upregulated (**Supplementary Figures 6G–I**).

### 3.10 YKT6 knockdown suppresses mTORC1 activity and induces cell-line-dependent feedback responses in the PI3K-AKT pathway

Based on LinkedOmics analysis, we identified genes that were highly correlated with YKT6 expression in breast cancer. By further surveying the literature, we found that several of these YKT6-associated genes have been reported to participate in regulation of the PI3K–AKT signaling pathway (17–19). On this basis, we selected this pathway for subsequent experimental validation. Given its central role in breast cancer progression, we further examined the activity of this pathway at the protein level. Because activation of this pathway is mainly controlled by post-translational phosphorylation rather than transcriptional changes, we evaluated its activity at the protein level. As shown in **Figure 7E**, YKT6 knockdown consistently reduced the phosphorylation of mTOR at Ser2448 in MCF-7, MDA-MB-231, and MDA-MB-468 cells, suggesting attenuation of mTORC1 signaling. In contrast, the upstream AKT response displayed cell-line-dependent differences. In MCF-7 cells, p-AKT (Ser473) levels were increased following YKT6 silencing (**Figure 7G**), which may reflect a compensatory response associated with mTORC1 inhibition. This effect was less evident in MDA-MB-231 cells (**Figure 7F**), whereas in MDA-MB-468 cells, p-AKT levels were reduced (**Figure 7H**).These findings suggest that YKT6 is associated with modulation of mTORC1 activity in breast cancer cells, while the regulation of upstream PI3K–AKT signaling may be context-dependent and differ among molecular subtypes.

## 4 Discussion

SNARE proteins are responsible for nearly all intracellular membrane fusion and exocytosis processes, which are essential for the exchange of substances between the cell and its environment (20, 21). Defects in these processes are associated with a variety of human diseases, including cancers. Increasing evidence suggests that the SNARE protein family plays a critical role in tumor progression, exhibiting abnormal expression in various cancers, and actively participating in tumor proliferation, invasion, metastasis, immune evasion, and therapeutic resistance (22–25). Targeting SNARE proteins may help delay tumor progression (26).

As a member of the SNARE family, YKT6 has been found to be significantly upregulated in several tumor tissues and can act as a prognostic biomarker for cancer (16, 27–29). Our study further indicates that YKT6 may serve as an independent prognostic factor in breast cancer. The prognosis of breast cancer is determined by the complex interplay among pathological subtypes, molecular driver events, host-related factors, and the tumor microenvironment. A recent investigation of germline mutation profiles in an Indian TNBC cohort delineated population-specific germline alterations and their potential clinical implications, highlighting the importance of genetic background in shaping tumor behavior and clinical heterogeneity across subtypes (30). In addition, transcriptomic analyses have shown that several frequently mutated genes in breast cancer, including PIK3CA, PTEN, RUNX1, and FAT1, display differential expression patterns according to menopausal status and hormone receptor context, suggesting that age and endocrine milieu modulate oncogenic programs (31).

In the present study, high YKT6 expression was associated with elevated tumor mutational burden and enrichment of TP53 mutations, implying a potential link between YKT6 and genomic instability as well as aggressive tumor evolution. TP53 is among the most pivotal driver genes in breast cancer, and its inactivation promotes cell-cycle dysregulation, enhanced metastatic capacity, and therapeutic resistance. The coexistence of YKT6 overexpression with TP53 alterations may therefore define a subgroup of breast cancers with increased proliferative and invasive potential. These findings suggest that YKT6 is not merely a passive biomarker but may participate in oncogenic networks that cooperate with canonical driver events. Although TP53 mutations were more frequent in tumors with high YKT6 expression, the present study does not establish a mechanistic dependency between these two events. Future studies will be required to determine whether YKT6 acts upstream, downstream, or independently of TP53-driven oncogenic processes.

Beyond molecular drivers, pathological features related to invasion and dissemination also exert a major influence on prognosis. Tumor budding, characterized by small clusters of cells detached from the main tumor mass, has been closely associated with lymphovascular invasion, lymph node metastasis, and aggressive behavior (32). Our functional assays demonstrated that YKT6 promotes breast cancer cell proliferation and invasion, supporting the notion that YKT6 may contribute to cellular programs underlying metastatic spread rather than representing only an *in vitro* phenotype. This observation is consistent with previous findings in lung adenocarcinoma (27).

The tumor immune microenvironment is likewise a critical determinant of therapeutic response. Prior studies in non–small cell lung cancer have reported a positive association between YKT6 expression and an immunosuppressive phenotype in tumor cell infiltration–negative draining lymph nodes (33). Beyond the classical PD-1/PD-L1 axis, tumor progression is further regulated by higher-order ecosystem interactions among immune cells and tumor-associated microbiota. The immune–oncology–microbiome (IOM) triad proposes that intratumoral microbiomes influence cancer evolution and treatment response by modulating immune evasion, genomic instability, and inflammatory signaling (34). Moreover, recent TNBC studies emphasize that immunotherapy should be guided by biomarker-based stratification, particularly in neoadjuvant and elderly populations where efficacy must be balanced against toxicity (35) (36). In parallel, gene signature–based drug screening has identified candidate agents capable of reversing myeloid-derived suppressor cell (MDSC)–mediated immunosuppression and enhancing immune checkpoint efficacy (37). In this context, our data show that YKT6 expression showed potential associations with immune cell infiltration across multiple computational methods (TIMER, CIBERSORT, and ssGSEA). However, these associations were generally modest, and sensitivity analyses indicated that some correlations were attenuated after excluding extreme values, suggesting a potential influence of outliers. In addition, YKT6-high breast tumors are preferentially associated with TIM-3 rather than the PD-1/PD-L1 axis, suggesting that YKT6 may participate in non-classical immunosuppressive pathways. Given that both HAVCR2 are increasingly recognized as key compensatory checkpoints in tumors resistant to PD-1/PD-L1 blockade (38), patients with high YKT6 expression may be more suitable for therapeutic strategies targeting alternative inhibitory checkpoints. Overall, these findings suggest that YKT6 may be associated with the tumor immune microenvironment, although further experimental studies are required to clarify its functional role. Furthermore, the differential responses observed after YKT6 knockdown in MCF-7, MDA-MB-231, and MDA-MB-468 cells further underscore the context-dependent roles of YKT6 across luminal and triple-negative breast cancer subtypes.

While prior studies have reported an association between YKT6 expression and chemoresistance in breast cancer (39), our study extends these observations by integrating large-scale multivariate survival analyses with systematic functional validation. Our results indicate that YKT6 functions as an independent prognostic factor and is associated with aggressive and metastatic phenotypes in breast cancer. In addition, our analysis of immune infiltration patterns provides a previously underexplored dimension of YKT6 biology, suggesting a potential link between YKT6 expression and the tumor immune microenvironment.

Taken together, YKT6 status may not only reflect tumor aggressiveness but also provide a rationale for integrating pathway-targeted inhibition with immune modulation in future therapeutic strategies.

Several limitations of the present study should be acknowledged. First, *in vivo* studies and rescue experiments will be needed to further evaluate the role of YKT6 in breast cancer invasion and metastasis. Second, the transcriptomic profiling was mainly performed in a single representative breast cancer cell line. Future studies with optimized experimental conditions and additional cell models will be required to further validate the YKT6-associated transcriptional programs. Third, migration was assessed using wound-healing assays under serum-free conditions to minimize proliferation effects. However, in MCF-7 cells, reduced proliferation may partially contribute to the migration phenotype. To better isolate the direct effects of YKT6 on cell motility, more refined migration assays, such as spheroid, organoid, microfluidic, and organ-on-a-chip platforms, which provide more physiologically relevant conditions, could be employed in future studies (40). Finally, the impact of YKT6 knockdown on mTOR signaling was mainly inferred from changes in phosphorylation of pathway components in a limited number of cell lines. Although a consistent trend toward reduced mTORC1 activity was observed, the upstream AKT response varied across breast cancer subtypes, suggesting that these effects may be context-dependent and not universally generalizable. The precise biochemical granularity and the direct molecular ‘interactome’ governing these effects remain to be fully elucidated. Future studies should investigate the potential impact of YKT6 in low-expression epithelial contexts to more fully understand the physiological and pathological roles of YKT6.

## 5 Conclusion

YKT6 is overexpressed in breast cancer tissues and can serve as a prognostic predictor for breast cancer patients. The immune infiltration analysis indicates that high YKT6 expression is likely related to an immunosuppressive microenvironment, offering a potential new therapeutic target for cancer immunotherapy.

## Data Availability

The original contributions presented in the study are included in the article/**Supplementary Material**. Further inquiries can be directed to the corresponding author.
