# Supplementary material for: YKT6 promotes breast cancer progression and is associated with poor prognosis and immune infiltration
Source: Front Immunol. 2026 Apr 22;17:1742661. doi: 10.3389/fimmu.2026.1742661 (PMC13144055; doi:10.3389/fimmu.2026.1742661)
Supplement: Supplementary file 1 [file DataSheet1.pdf]

Supplementary Material

YKT6 Promotes Breast Cancer Progression and Is Associated with Poor Prognosis and Immune Infiltration

Meilin Zhang, Jinjin Yuan, Yaxuan Liu, Yiran Qiu, Mindi Zhang, Hongliang Chen*

* Correspondence:

Corresponding Author

13671852284@163.com

1.1 Supplementary Figures

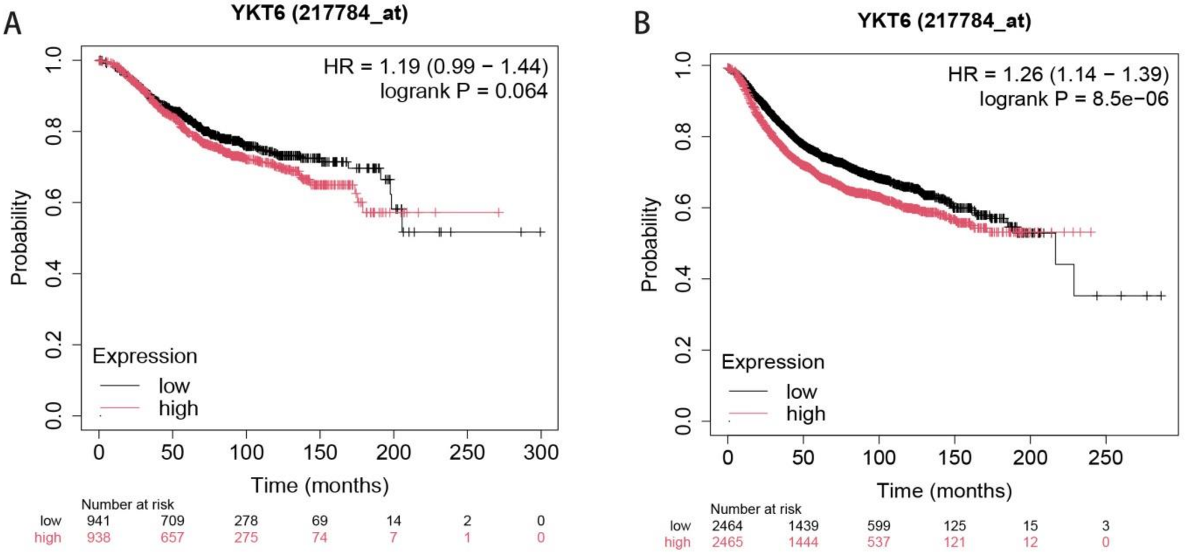

Supplementary Figure 1. High YKT6 expression is associated with poor survival outcomes in breast cancer :KM Plotter Database Analysis (A) Association of YKT6 Expression with Overall Survival (OS) in Breast Cancer: KM Plotter Database Analysis (B) Association of YKT6 Expression with Relapse-Free Survival (RFS) in Breast Cancer: KM Plotter Database Analysis

A

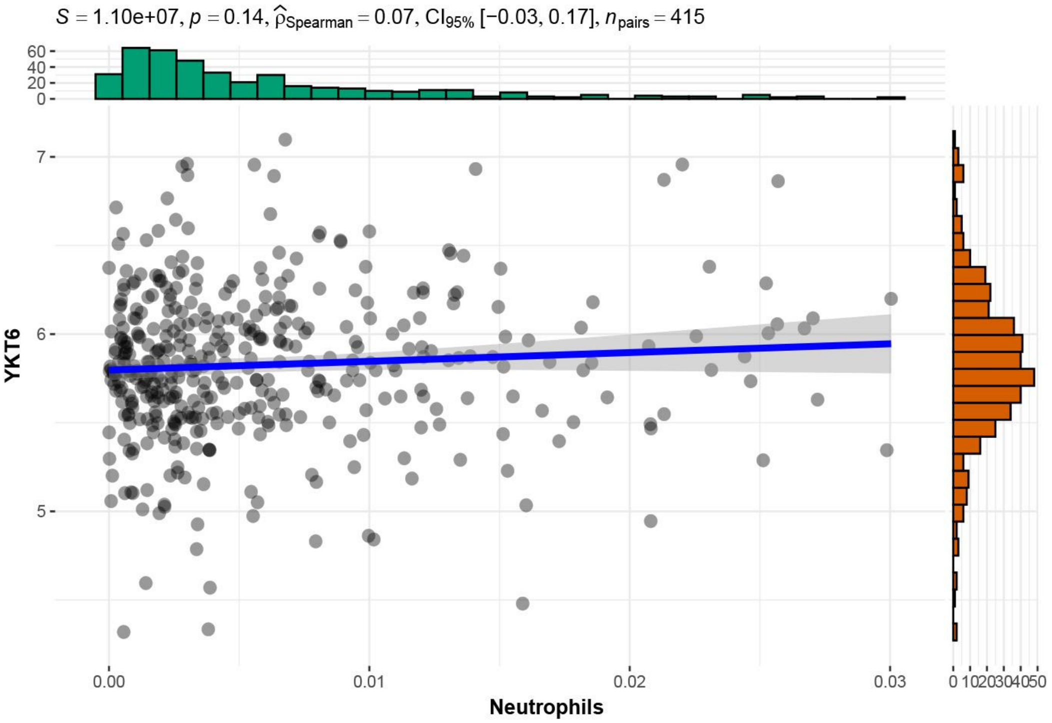

Supplementary Figure 2. (A)Sensitivity analysis of correlation between YKT6 and neutrophil infiltration after excluding extreme values (1% tails).

A

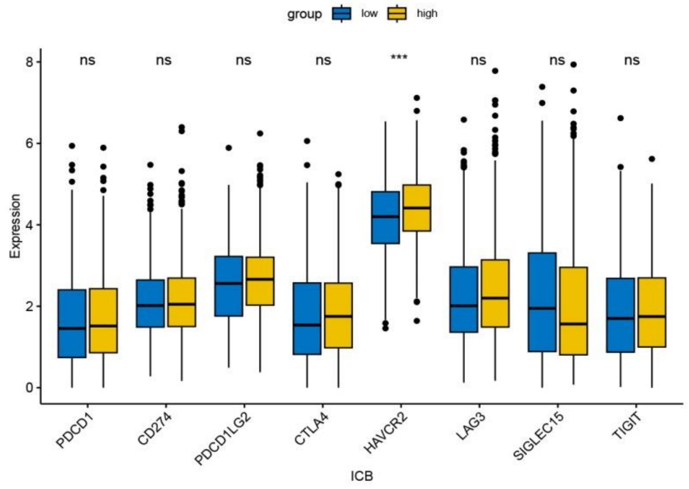

Supplementary Figure 3. (A) Immune Checkpoint Correlations in high and low YKT6 expression breast cancer patients.

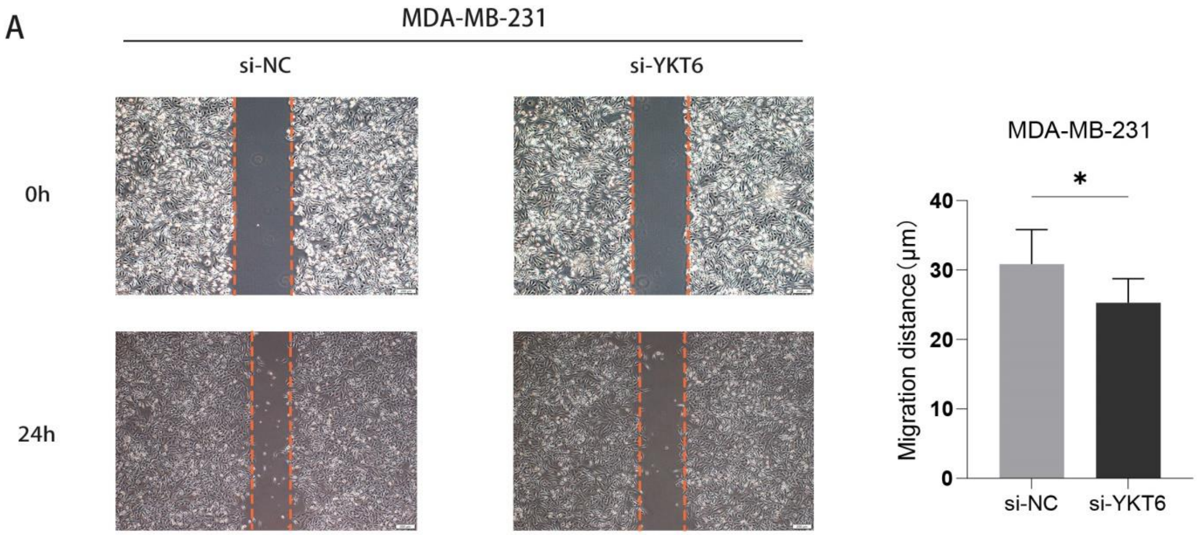

Supplementary Figure 4. (A) Wound-healing (scratch) assay in MDA-MB-231 cells. Cell migratory capacity was evaluated at 24 h in MDA-MB-231 cells transfected with si-NC or si-YKT6.

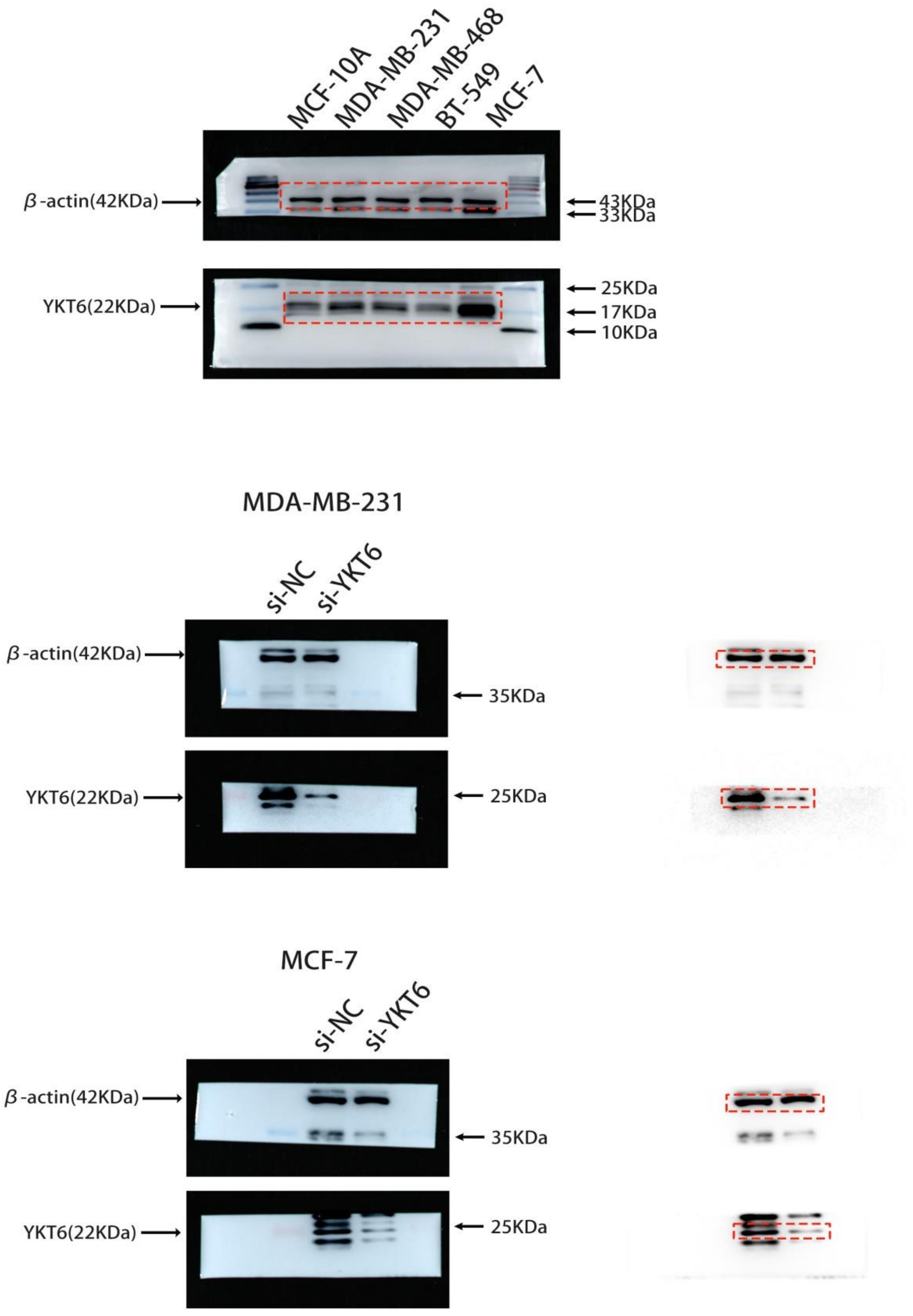

Supplementary Figure 5. Original, uncropped Western blot membranes corresponding to the representative blots shown in Figures 5A and D

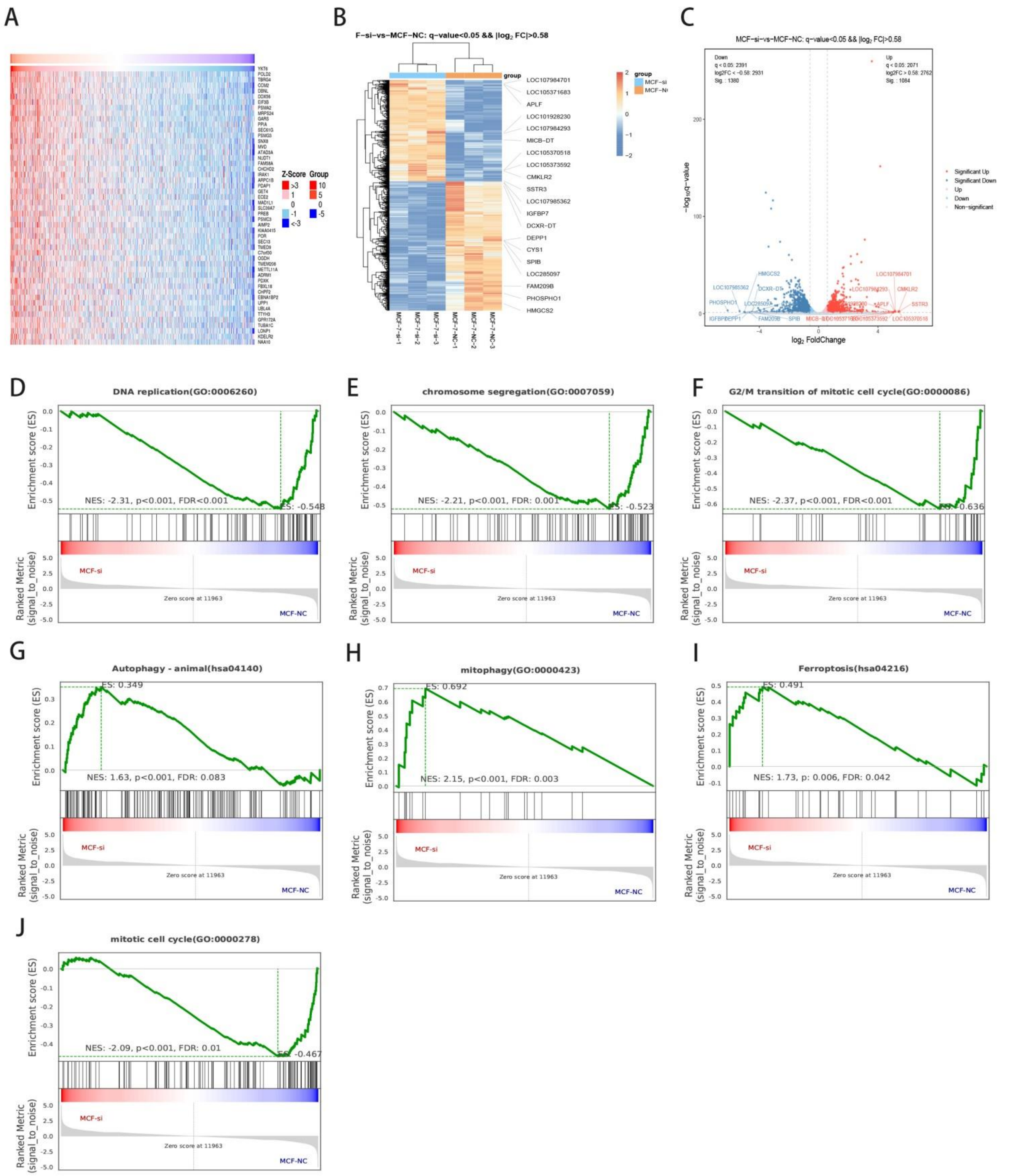

Supplementary Figure 6. RNA-Seq Analysis of YKT6-Regulated Molecular Pathways : (A) Heat maps showing TOP 50 genes positively related to YKT6 : LinkedOmics Database (B) Heatmap plot showed differentially expressed genes in cells with YKT6 knockdown as compared with control cells. (C) Volcano plot showed differentially expressed genes in cells with YKT6 knockdown as compared with control cells. (D) GSEA shows inhibition of DNA replication progression following YKT6

depletion. (E) GSEA shows inhibition of chromosome segregation progression following YKT6 depletion. (F) GSEA shows inhibition of G2/M transition of the mitotic cell cycle progression following YKT6 depletion. (G) GSEA shows inhibition of autophagy progression following YKT6 depletion. (H) GSEA shows inhibition of mitophagy progression following YKT6 depletion. (I) GSEA shows inhibition of ferroptosis progression following YKT6 depletion. (J) GSEA shows inhibition of mitotic cell cycle progression following YKT6 depletion

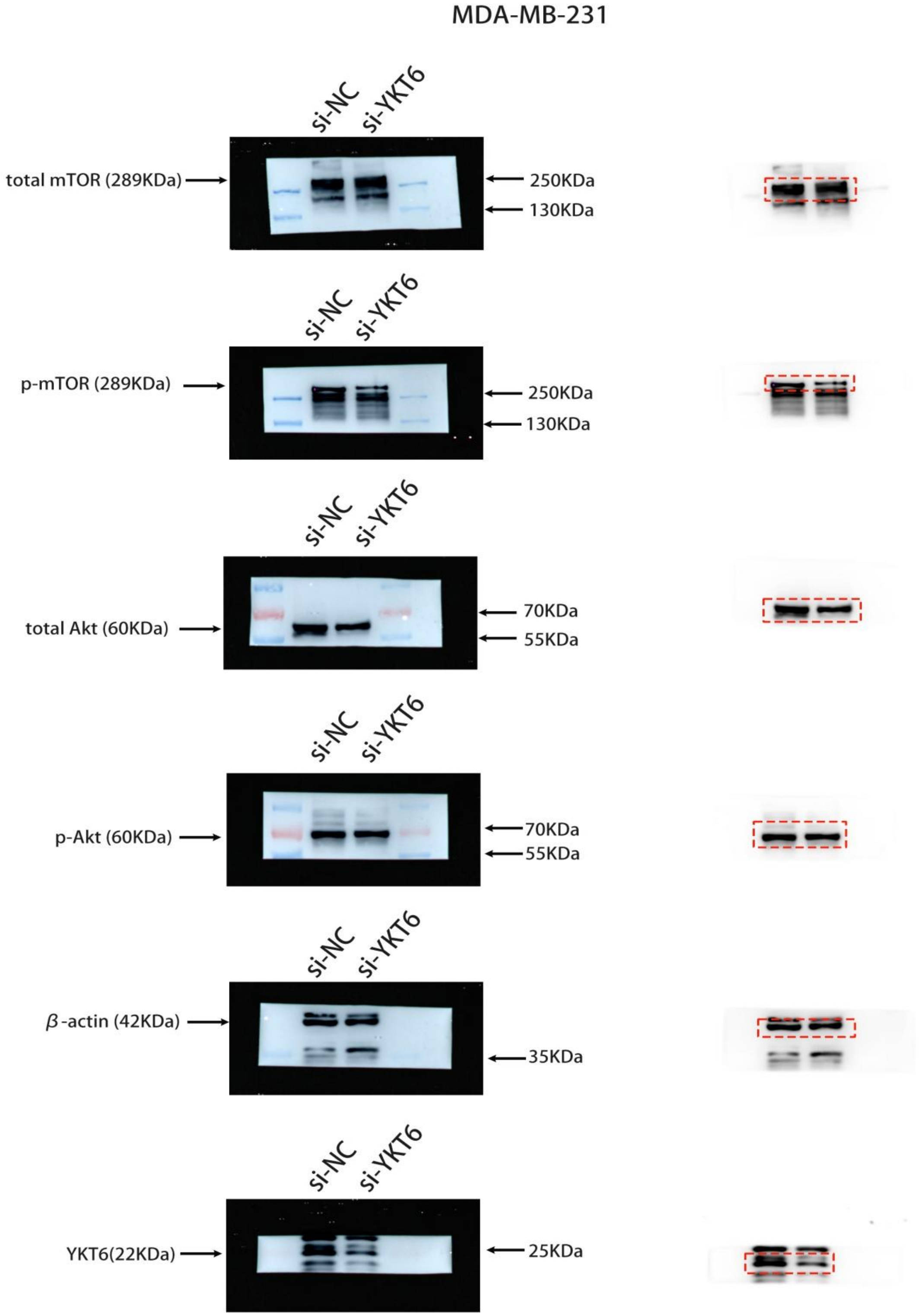

Supplementary Figure 7. Original, uncropped Western blot membranes corresponding to the representative blots shown in Figures 7E

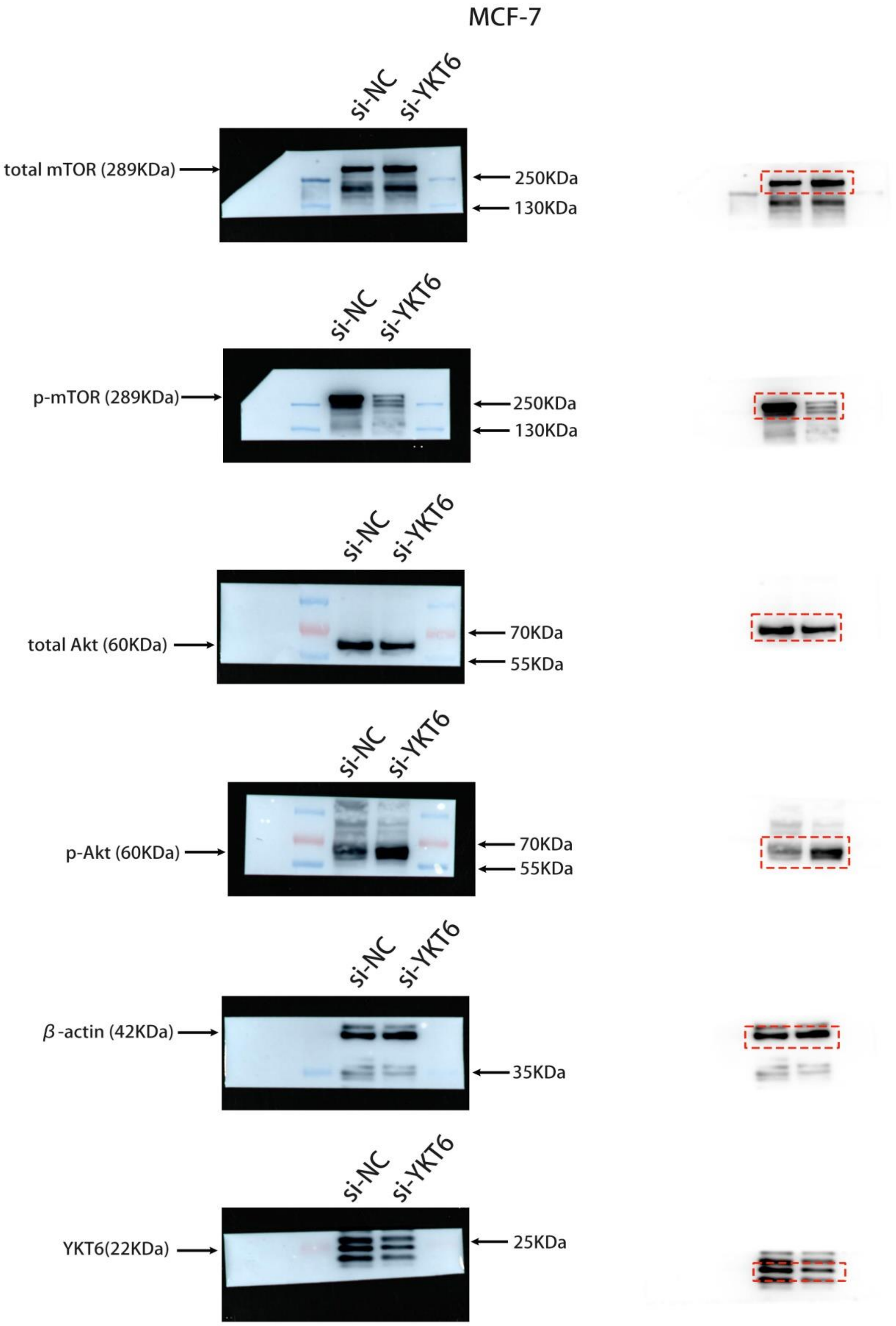

Supplementary Figure 8. Original, uncropped Western blot membranes corresponding to the representative blots shown in Figures 7E

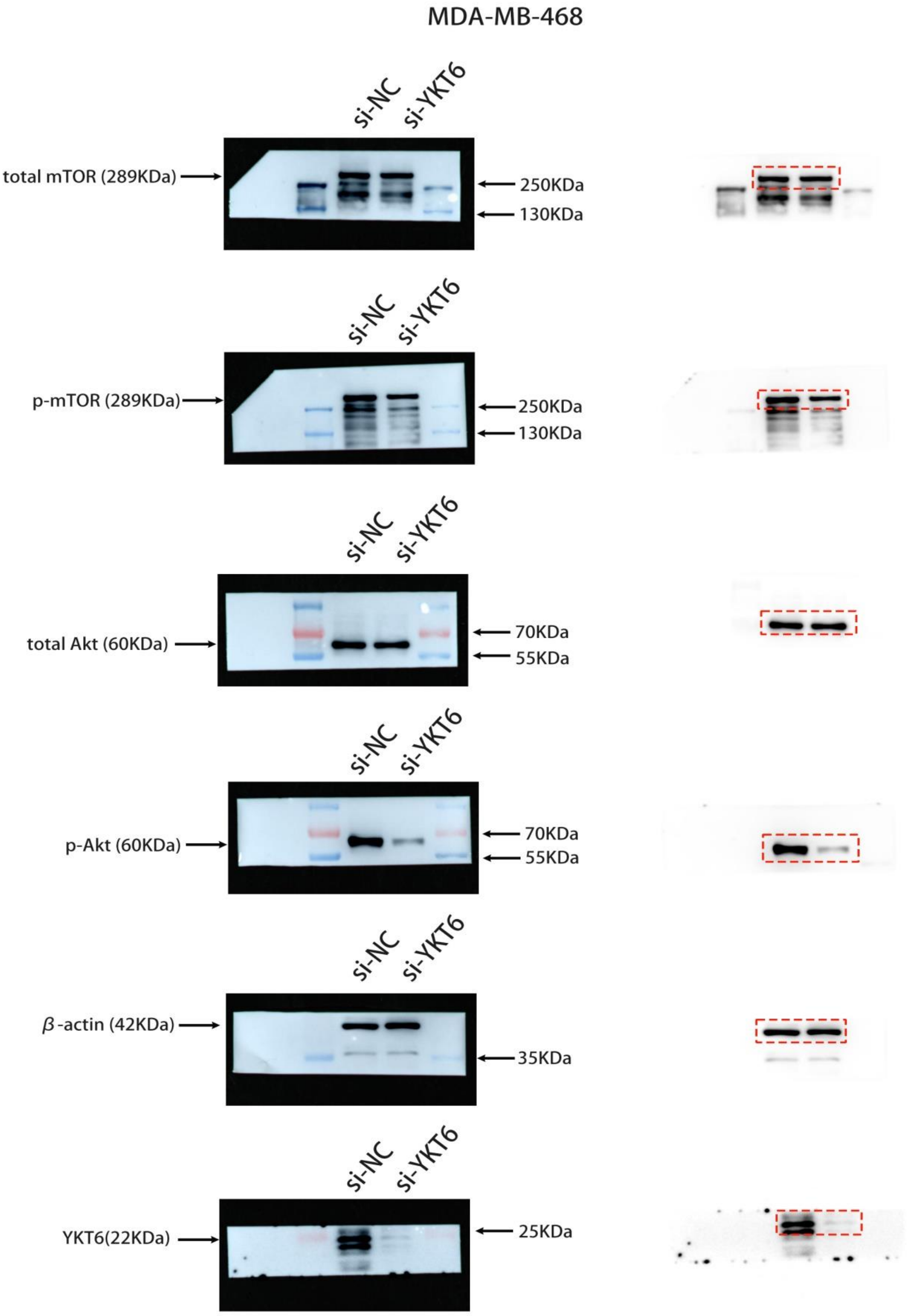

Supplementary Figure 9. Original, uncropped Western blot membranes corresponding to the representative blots shown in Figures 7E
